# Transplanting severely obese cirrhotic patients: Heavy clouds still on the horizon

**DOI:** 10.1002/ueg2.12234

**Published:** 2022-04-26

**Authors:** Patrizia Burra, Alberto Ferrarese

**Affiliations:** ^1^ Multivisceral Transplant Unit, Department of Surgery, Oncology and Gastroenterology Padua University Hospital Padua Italy; ^2^ Gastroenterology Verona University Hospital, Ospedale Borgo Trento Verona Italy

Obesity represents one of the major epidemiologic challenges of this century, causing more than 300,000 premature deaths per year, increased healthcare costs and social stigma.^1^ More than 75% of obese and >90% severely obese people develop non‐alcoholic fatty liver disease,^2^ which in turn becomes a strong risk factor for liver fibrosis, cirrhosis and hepatocellular carcinoma. This obesity epidemic is therefore also showing its strong effects in the liver transplant (LT) setting, with an increasing proportion of obese or morbidly obese potential LT candidates.

Until now, LT has been regularly offered to class I obesity recipients (BMI < 35), whereas class II obesity ones (35 ≤ BMI < 40) are given a thorough multidisciplinary evaluation. Although the high intra, peri and post‐operative complication rates make class III obesity (BMI ≥ 40) a relative contraindication to LT,^3^, ^4^ an increasing body of evidence is emerging for such morbidly obese candidates, stretching the current boundaries. Indeed, since class III obesity represents a risk factor for the development of acute‐on‐chronic liver failure, waitlist drop‐out and mortality,^5^ but it does not always translate into inferior post‐operative outcomes,^6^, ^7^ and strictly selected patients may achieve high survival benefit after transplantation.

What happens to obese patients while waiting for a graft? It should be remembered that lifestyle modification through diet and physical exercise are the most powerful tool we can offer up to now, since safe and effective medications are lacking in clinical practice. Moreover, pre‐transplant bariatric surgery (mainly laparoscopic sleeve gastrectomy) may be an option for compensated cirrhotic patients, or should be planned in combination with transplant in decompensated ones and in selected centers.^8^

Some answers to these questions come from the study by Delacote et al., published in the current issue of the *Journal*.^9^ The Authors prospectively evaluated 3427 obese patients (of whom 960 had a BMI ≥35) listed for LT in France over 10 years. The Authors demonstrated that patients with class II‐III obesity had a higher 1‐year probability of death/drop‐out from the waiting list, and less probability of being transplanted when compared to non‐obese patients. Conversely, class I obese candidates had similar waitlist outcomes to non‐obese recipients. When the Authors looked at the possible causes, it appeared that the number of proposals for a graft was the same for patients with BMI lower or higher than 35, but the number of refusals for size incompatibility was larger in the latter group. This was explained by the fear of a *small‐for‐size syndrome* after transplant, being highlighted more in males than in females (given the higher medium height, males are heavier at the same BMI, and suffer from a disadvantageous graft‐to‐recipient weight ratio).

Translating this message into clinical practice, physicians should advise severely obese (especially male) candidates that they may remain for a longer time on the waiting list, with a higher risk of death/drop‐out. It remains to be established if these findings can be confirmed in other countries (where the prevalence of severe obesity is different), or if the utilization of grafts from strictly selected morbidly obese donors may reduce this imbalance.^10^

Although this study has merit, some important questions remain unsolved. First, obesity was assessed only at time of waitlisting using the BMI: this can change over time, at least for the group of patients who follow a medically supervised weight loss program, or undergo bariatric surgery, and for those who remain on the waiting list for a long time. Second, the study depicted the waiting list course, without adding information about causes of death/drop‐out, as well as the post‐LT survival (and transplant benefit). Moreover, it could be helpful to understand if lifestyle non‐adherence or further weight gain have been considered as drop‐out criteria from the waiting list. Third, obesity is a complex syndrome needing a multidimensional assessment. BMI, which may be influenced by fluid retention in LT candidates, is a surrogate marker that we continue to use for obesity classification in clinical practice. It should be associated with other parameters, able to define the presence of sarcopenia, myosteatosis, type and grade of chronic inflammation, which probably act as further risk factors of decompensation and death, over obesity itself.^11^ In summary, we congratulate Delacote et al.^9^ on advancing knowledge with their thorough study: *heavy* clouds still hang in the sky of liver transplantation for severely obese cirrhotic patients. Therefore, more efforts are urgently needed by Scientific Societies to properly classify obesity ‐ going beyond the BMI—and identify those candidates who may achieve the highest survival benefit from transplant, taking into consideration the severity of liver disease, other pathological features, associated morbidities, adherence of patients to a weight loss program and overcome the stigma associated with obesity, especially in the female population.

## CONFLICT OF INTEREST

The authors have none to declare regarding this manuscript.

## AUTHOR CONTRIBUTION

All the authors contributed to conceptualization, writing and revising the manuscript. They approved the final version of the manuscript.

## Data Availability

Data sharing is not applicable to this article as no new data were created or analyzed in this study.
